# Understanding inequalities in the coverage of adolescent sexual and reproductive health services: a qualitative case study of the selected regions of Zambia

**DOI:** 10.3389/frph.2024.1399289

**Published:** 2024-08-06

**Authors:** Margarate Nzala Munakampe, Alice Ngoma-Hazemba, Mutale Sampa, Choolwe N. Jacobs

**Affiliations:** ^1^ Department of Health Policy and Management, School of Public Health, University of Zambia, Lusaka, Zambia; ^2^ Yakini Health Research Institute, Lusaka, Zambia; ^3^ Women in Global Health, Zambia Chapter, Lusaka, Zambia; ^4^ Department of Community and Family Medicine, School of Public Health, University of Zambia, Lusaka, Zambia; ^5^ Department of Epidemiology and Biostatistics, School of Public Health, University of Zambia, Lusaka, Zambia

**Keywords:** acceptability, accessibility, availability, quality, adolescents and young people, sexual and reproductive health, human rights framework

## Abstract

**Introduction:**

Despite substantial investment in improving healthcare among adolescents in low- and middle-income countries, barriers to access and utilization of services persist, especially to sexual and reproductive health (SRH) services. In response to adolescents' health service needs due to their vulnerability, interventions aimed at improving access and utilization of sexual and reproductive health services have been implemented in specific regions of Zambia. To highlight progress in the access and the overall delivery of services in Zambia, in the wake of a system-level funding mechanism, this paper aims to understand the accessibility, availability, acceptability and quality (AAAQ) of health services provided to young people.

**Materials and methods:**

In a qualitative case study, 48 discussions- 32 individual interviews with stakeholders and 16 focus group discussions, consisting of 128 male and female adolescents were conducted in six districts from Eastern, Southern and Muchinga provinces of Zambia. Interviews were audio-recorded, recordings transcribed verbatim, and transcripts were analysed using deductive thematic analysis, using the AAAQ framework and Atun's framework on integration, as a guide to reporting the findings.

**Results:**

We found that adolescents knew of and had access to common commodities and services- male condoms, health education and HIV counselling and testing. However, availability was affected by access-related barriers such as frequent stock-outs and insufficiently trained healthcare providers. In addition, accessibility was more restricted during the COVID-19 pandemic lockdown and compounded by the low acceptability of SRH service among adolescents across all contexts. This led to the use of alternatives such as herbal medicine and maintained common myths and misconceptions. The overall quality was marred by the lack of dedicated spaces for adolescent health services and the lack of information, education and communication (IEC) materials in some spaces.

**Conclusion:**

While it was noted that some services were available for adolescents in all the study sites, numerous barriers inhibited access to these services and had an impact on the quality-of-service provision. With the added restriction to SRH service asses for young people, due to the low acceptability of adolescent SRH service use, the overall integration of adolescent SRH interventions into routine service provision was low and can be improved by targeting contextual barriers and maintaining best practices.

## Introduction

Despite efforts to accelerate progress in the coverage of essential health services, some populations remain “left behind” (1). Attaining the United Nations 2030 Agenda for Sustainable Development and the Sustainable Development Goals (SDGs) requires protective laws, policies and practices that promote non-discrimination, equality and non-violence towards vulnerable and key populations -sex workers, gay men and other men who have sex with men, transgender people, people who inject drugs, indigenous populations, internally displaced populations, people in prisons and other enclosed settings and *adolescents and young people*, and other people that have been left behind (2).

The goals of Universal Health Coverage (UHC) are reinforced by the need to provide prevention, promotion, treatment, rehabilitation and palliative services to all who need them (3). In addition, providing sexual and reproductive health (SRH) services to all, including adolescents and young people (AYP) remains a major priority for achieving global UHC (4), and this is necessary to “ensure universal access to sexual and reproductive health-care services, including for family planning, information and education, and the integration of reproductive health into national strategies and programmes by 2030” -SGD target 3.7 (5).

Adolescent Sexual and Reproductive Health and Rights (ASRHR), according to the World Health Organization (WHO), is “a state of complete physical, mental and social well-being, not merely the absence of disease and infirmity, in all matters relating to the reproductive system and its functions and processes specifically applied to adolescents” (6). Across the globe, more than 16 million young women (15–19 years old) residing in low- & middle-income countries give birth yearly (7). This trend worsens in the sub-Saharan context, where the prevalence of teen pregnancy is as high as 18.8%, and SRH issues remain highly contested, particularly concerning AYP (8). This usually results in barriers to access and utilization of SRH services by AYP, rooted in health worker bias, the lack of willingness from health workers to provide services, socio-economic constraints, or the lack of knowledge about sexual health needs (9). This has left AYP more vulnerable to “illegal” or “unsafe” options (10) and other negative health consequences such as the unmet need for contraception, harmful gender inequalities, high sexual gender-based violence (SGBV) rates, and alcohol and drug use disorders (11).

The Zambian government committed to improving adolescent health through policies such as the SRH focused on Reproductive, Maternal, Newborn, Child and Adolescent Health and Nutrition Communication and Advocacy Strategy 2018–2021, which aimed “to effectively target and serve adolescents and youth with quality accessible sexual and reproductive health information and services in and out of school” (12). These reinforced the need to protect young people from the negative effects of poor access to SRH services and an overall improvement in health status. However, coverage remained lacking as studies have shown variations in the availability, accessibility, acceptability and quality of interventions- negative healthcare workers attitudes, low access to services, poor infrastructure for adolescent SRH services, low contraceptive uptake, increased risk of contracting HIV as STIs, among others (13–16).

### The SIDA interventions

Within national level guidance, and to increase the availability and readiness of quality, increasing demand and uptake of physically, culturally, and financially accessible health services, the Zambian Ministry of Health, with support the from Swedish International Development Cooperation Agency (SIDA) supported SRH and nutrition services in four provinces of Zambia. SIDA-supported evidence-based interventions aimed to strengthen the health system at the national and sub-national levels as necessary to provide an enabling environment for effective reproductive, maternal, neonatal, child, adolescent health and nutrition (RMNCAH&N) service delivery. This was done by; (a) recruiting additional health workers; (b) capacity-building through training of health care providers, including provision of incentives for health care providers at various levels; (c) rehabilitating or constructing essential buildings; (d) purchasing different types of materials in the provinces; (e) key demand-creation activities for users; (f) several activities at the national level to contribute towards health systems strengthening; and (g) forming a steering committee to support monitoring of interventions across contexts. SIDA supported system-level interventions, which were all evidence-based interventions (EBI). Thus, no new interventions was rolled out, however, funding was made more available for existing interventions. More information on the nature of SIDA funding support and the interventions is available elsewhere (13, 17).

In understanding the effectiveness of investment in the health of a marginalised group such as AYP, equity becomes a key consideration (18). As such, the Human Rights-Based AAAQ Framework illuminates the short and long-term impact of such interventions, in trying to understand how young people claim their right to health care (19). Additionally, Atun's Integration Framework is useful in exploring the nature and extent of integration of the interventions into routine healthcare provision- speaking to questions of sustainment within the system. While there is no commonly accepted definition of “integration”, Atun et al., propose that “*(…) the adoption and diffusion of new health interventions and the extent to which they are integrated into critical health system functions will be influenced by the nature of the problem being addressed, the intervention, the adoption system, the health system characteristics, and the broad context*” (20).

Thus, integration can be influenced by the nature of the (i) problem being addressed, (i) the intervention, (iii) the adoption system, (iv) the health system characteristics, and (v) the broad context. This paper, therefore, aims to assess the availability, accessibility, acceptability and quality of care of adolescent SRH services in selected contexts of Zambia, and to ascertain the extent to which the intended interventions strengthened the health system as necessary to provide an enabling environment for effective RMNCAH&N service delivery, especially for young people. Understanding the inequalities of SRH services among adolescents is crucial for increasing access to services and utilization, and for empowering young people with their right to health. This is important due to the large number of adolescents in Zambia (60%) and the added benefit of investing in the population at this time of their lives.

## Materials and methods

### Design

This paper uses a qualitative descriptive case study design (in a constructionist paradigm), answering the “why” and “how” questions in the context of identified inequalities in the coverage of services (18), in some selected low-performing and well-performing districts with a focus on availability, accessibility, acceptability and quality of care (19) for RMNCAH&N interventions. This study design is suitable for understanding circumstances surrounding the uneven coverage of services for adolescents, in the real-world setting.

### Study setting

The study setting was selected districts from Eastern, Southern and Muchinga Provinces of Zambia. These districts included Choma, and Livingstone in the Southern province, Chinsali and Nakonde in Muchinga province, and Chipata and Mambwe in the Eastern province. The districts were purposively selected among the districts implementing RMNCAH&N interventions with support from SIDA in the three provinces, as guided by program implementers. Preliminary analysis of national-level coverage revealed that one of the provinces reported higher RMNCAH&N indicators, while another reported lower RMNCAH&N coverage indicators. The third province reported both high and low coverage indicators. Additionally, the analysis also showed improvements in rural settings, and poorer health outcomes in the urban areas. This analysis informed the selection of districts in each of the provinces. Thus, participants were drawn from the communities within the catchment areas of one rural and one urban public facility in each of the selected well or poor-performing districts.

### Study population and characteristics

The views expressed in this study were drawn from discussions with adolescent males and females, frontline healthcare providers, community health workers, traditional leaders and program staff at provincial or district health offices. Adolescent boys and girls were aged between 10 and 19 years, from whom consent (and assent where necessary) was obtained/provided and were living within the communities at least for a year preceding the interviews were enrolled. Within the community, the leaders interviewed included headmen or chiefs within the catchment area of the health facility, as well as civic leaders in the districts.

On the health care system side, frontline health care providers providing RMNCAH&N services at the health facility were enrolled; preferably those who received additional training in the Elimination of Mother-to-Child Transmission of HIV (eMTCT), Post-Abortion Care (PAC), Emergency Obstetric and Newborn Care (EmONC**)**, and Basic Emergency Obstetric and Newborn Care (BEmONC). Program staff- individuals involved in the management and coordination of the RMNCAH&N interventions, and preferably, those who had been working in their capacities since the first year of the project were included as they support the activities of the frontline healthcare workers. At the community level, Community Health Workers (CHWs), who included those volunteering in RMNCAH&N services, as well as CHWs supporting RMNCAH&N services at the health facility or in the community; and were living within the community of interest for not less than a year were enrolled into the study.

### Recruitment of participants

Community Health Volunteers (CHVs), mainly the Safe Motherhood Action Groups (SMAGs), facilitated the recruitment of participants for the study. Other CHWs from the Neighbourhood Health Committees aided recruitment. The SMAGs were trained to support women during their pregnancy and help to disseminate information about pregnancy, childbirth and family planning to create awareness of maternal and newborn health services, including adolescent health and nutrition. The SMAGs and CHWs interact with and live within the communities, as such, they were more helpful in identifying study participants particularly those that were not easy to locate by the research teams. The study team discussed the inclusion and exclusion criteria with the community volunteers, and they helped the study team to identify the required respondents.

### Sampling considerations

A heterogeneous selection strategy of participants of at least four (4) focus group discussions (FGDs) with adolescent males and females was drawn in each district (total of 16 FGDs). In addition, one (1) interview with a community leader and four (4) interviews with programme managers/health workers/community health volunteers were done in each sub-region (total of 32 interviews). Table 1 summarises the list of interviews that were conducted.

**Table 1:** List of participants in the study.

Type of participants	Approach^a^	Respondents (age range)	Number of sessions/participants per session	Total participants
Adolescent males and females	FGDs	Males (10–19) Median age-17	8 sessions/8 participants	64
		Females (10–19) Median age-16	8 sessions/8 participants	64
Key informers	IDIs	District or Provincial Office	12 sessions/1 participant	12
Frontline healthcare providers	IDIs	Programme managers, implementers	8 sessions/1 participant	8
Community health workers	IDIs	SMAGs, counsellors, treatment supporters	8 sessions/1 participant	8
Community leaders	IDIs	Chiefs or headmen	4 sessions/1 participant	4
		Total	48	160

^a^ Data collection approach; (IDI) in-depth interview or (FGD) focus-group discussion.

### Data collection

Data was collected in November 2020. All discussions-in-depth interviews (IDIs), key-informant interviews (KIIs), and focus-group discussions (FGDs) were conducted in private areas to ensure privacy and confidentiality and using semi-structured discussion guides to explore availability, accessibility, acceptability and quality of services. The discussion guides contained questions about family planning, STIs/HIV/AIDs prevention and treatment, antenatal care, nutrition services and youth-friendly services. The FGDs each included 8 participants, and discussions were held in locations agreed upon by the participants, within the communities and outside health facilities. The seating arrangement of participants during the FGDs was U-shaped to ensure interaction and for the moderator to easily observe everyone.

All interviews and discussions were conducted in English or the common local languages (such as Nyanja, Tonga or Bemba) in each region visited, or a combination of two or three of these. If participants gave consent, the interviews were digitally recorded. All interview guides (initially in English) were translated into Bemba, Nyanja and Tonga independently. To achieve data triangulation, interviews were conducted with different groups of participants from those included in the FGDs, using a semi-structured discussion guide. Both FGDs and IDIs were used for cross-verification and complementary purposes.

### Data management and analysis

The audio recordings of interviews were transcribed verbatim from either of the local languages used and then translated into English by professional transcribers/translators. A thematic analysis was used to put into context the findings of the study (21). A few (10%) transcripts were thoroughly read for familiarization and re-read by two independent researchers to develop an initial coding scheme. The codes agreed upon were organized to create categories or sub-themes and later main themes. Thereafter, all the transcripts were transferred into NVivo version 11 and were coded. The coding process allowed for the identification of emerging themes and subthemes. Finally, a report of the relationships between themes was presented.

### Quality control & data storage

The tools used to collect the data were pre-tested before data collection to identify potential deficiencies and to ensure consistency in understanding the research aims among data collection teams. All researchers involved in the study went out to one district (not one of the study districts) to collect data using the tools, and this process was discussed during a training session. All the research assistants were trained in data collection and familiarised themselves with the tools. The community mobilizers were also trained to screen the participants to confirm that they met the eligibility criteria. About ten per cent of the recorded interviews/discussions were transcribed verbatim in the local languages used and then translated into English by research assistants. Some transcripts (10) were randomly selected and verified by a back translation into Bemba, Tonga or Nyanja for accuracy. Transcripts that were not back-translated were reviewed by listening to the original voice recordings to ensure that they retained the original meanings after translation. All the data collected and the related files including field notes were stored on password-protected hard drives and all access to the data was restricted to the project staff. The data will be stored for a period of up to five years from data collection, after which it will be destroyed.

### Ethical considerations

Ethical clearance for the study was granted by the University of Zambia Biomedical Research Ethics Committee (REF 1236-2020) and the National Health Research Authority. The researchers administered informed consent, conducted interviews and facilitated/moderated discussions in private places to ensure confidentiality. Personal identifiers such as names of participants were not recorded in the audio or any other documentation. For participants below the consenting age, both consent from parents and guardians and assent for them to take part in the study were sought. It was well noted that younger participants lacked agency and were at risk of being coerced into taking part in the study, thus, the assent process was emphasised to the researchers who administered it. In addition, the researchers' power and positions were actively addressed during training, to reduce biases.

#### Findings

This paper aims to assess the availability, accessibility, acceptability and quality of care of adolescent SRH services in selected provinces in Zambia, in line with the human rights-based AAAQ framework (19). In this section, we unpacked each of the availability, accessibility, and acceptability constructs (with quality included in each of them) to provide highlights of some of the conversations with intervention implementers and adolescents.

#### Availability

##### Available services

Participants described the available services for adolescents with caution because it is a sensitive subject to debate. Among the services mentioned were youth-friendly services that encompass condom distribution and contraception, sexual health education, dedicated ART services and a deliberate effort to support girls to go back to school after childbirth. Specific youth-friendly services reported to be available were antenatal care, family planning, treatment for STIs, HIV testing and counselling. The MCH Coordinator from a well-performing district said:

“(…) we provide almost all the services, such as antenatal care because we separate [adolescents from the adults], adolescents have their own day for antenatal, family planning, HIV testing. They also go out to sensitize the community; they visit schools to sensitize on adolescent activities or services that are offered from the clinics (…)”

[MCH Coordinator from a well-performing district].

##### Health provider as a barrier to availability

While the youth-friendly services were advocated for by providers, the fear of creating an access barrier in case the adolescents recognized the coordinator as a parent as well, a young healthcare provider was assigned to coordinate the services for adolescents, while the MCH coordinator remained to oversee the implementation. According to an MCH Coordinator, from a well-performing district,

“(…) when they come for family planning and they find me who is the friend to the mother, do you think this fourteen-year-old will be free to get family planning. She will think that I going to tell her mother (…). Even here we have got an adolescent focal person … someone who looks younger because I used to coordinate but I said I think I need to shift to a younger man to handle the services…I am just a coordinator am not an adolescent, I don't face adolescents in the office (…)”

[MCH Coordinator, from a well-performing district].

##### Knowledge of contraceptive methods

Knowledge of methods was linked to what was available. When asked if they knew any contraceptive methods, adolescents in rural low—performing districts mentioned injectables, Intrauterine Contraceptive Devices (IUCD) [copper was most prominent] and “morning-after” pills [emergency contraception]. Health education activities were reported in some form or the other, and in the rural and urban districts of the low-performing region, knowledge about a variety of FP methods and other SRH services was still widespread among the adolescents.

Respondent: Me, I heard that there are oral, oral meaning, (Pause) Tablets, Pills, then there is also long-term service, like *Jadelle* for 5 years and that one for 3 years (…).

Respondent: (…) *Depo*, there is for 2 months and 3 months. Again, they give a condom as part of family planning that prevents not only pregnancy but also STIs (…)

Respondent: …there is the one for pills, the family planning and that one for injections for years and loop, and condoms.

[Adolescent girls, urban, low-performing district]

##### Usage of contraceptive methods

Usage of methods was linked to what was available. In a high-performing province, adolescents were more inclined towards using condoms as a form of Family Planning (FP). In a rural subregion, a health facility in charge reported that they had dedicated days when they attended to adolescents living with HIV and accessing ART. Community leaders were not only involved in sex education but also advocating and encouraging girls who left school due to pregnancy to go back. A community leader in a rural well-performing district explained:

“…in my case, I call maybe two teachers, community members and girls dropped out of school because of pregnancies. The teachers advise them to go back to school since they had given birth and their children have grown up (…) so they are advised and even inspired by examples of people with children who went back to school (…)”

[Community leader in a rural well-performing district].

#### Accessibility

##### Accessible methods

In framing accessible adolescent health services, this section describes how the participants highlighted only what they understood in terms of health education activities, condom distribution and access, HIV counselling and testing. When considered against the narratives from the MCH coordinators, the DDH and health care providers, it is possible to contextualise them as barriers and facilitators to accessibility. Adolescents accessed family planning products such as condoms from the health facilities as well as shops: a female adolescent from a well-performing urban district said:

“We have no challenges. People are into groups, and they meet separately to combat the distance issue. Condoms are given away. Personally, I have collected condoms at this health facility. Others refuse to get condoms due to fear of being looked down upon”.

[Female adolescent from a well-performing urban district].

Other reproductive health services accessed by adolescents across contexts included HIV counselling and testing as well as cervical cancer screening.

##### Barriers to access: healthcare workers as barriers

However, some healthcare providers were still interrogating and discouraging youths from accessing condoms or contraception or even other reproductive health services such as HIV counselling and testing. While healthcare providers meant to encourage the treatment of both partners, they also prevented the use of services for young people who were proactive but did not follow all their guidelines. A female adolescent from a low-performing urban district said:

“On my part, I came to test for HIV. They said that I should come with my boyfriend. That is when I asked what [happens] if I did not have a boyfriend. [They asked if it] is he who has gotten sick or me? Then they said just in case I was having sex with my boyfriend while I had the disease, we [healthcare providers] do not know who gave it to you. Then I said now what if my boyfriend is in Lusaka and not here, they said you should look for another boyfriend and come with him”.

[A female adolescent from a low-performing urban district]

##### Herbal medicine as an alternative to restricted access

Regardless, adolescents had some access to information on the types and benefits of different contraception methods. Adolescent girls from low-performing rural areas described family planning in the context of the prevention of pregnancy and not diseases. However, information on the use of herbal medicine for family planning was equally mentioned as it was passed on to them in the community. Sexuality education was also taught in schools as reported across contexts. An adolescent male from a low-performing rural facility said: “*We learnt it from school, people used to come and teach us how to use family planning and how to get it at the clinic*”. A female adolescent from a low-performing urban district mentioned other sources of information and services related to family planning. She said

“(…) I was also taught at church (…) and even also hear from our friends in the community. Information is also accessed from radios at home, TVs and even phones (three-quarters of us have phones) of course it is not like these things are hidden, we can find them anywhere even on social media, on Facebook we do find these things”.

[Female adolescent from a low-performing urban district].

Other young people noted that sex education was not taught in their schools.

In the low-performing rural and urban regions, numerous barriers to access were noted. Most facilities are understaffed, and family planning services were only available on certain days, alongside other women in the community. While adolescents felt that, the community-based activities were helpful for most young people, particularly those who were scared to visit the facility or lived far away from it. A female adolescent from a low-performing rural district said: “*It can be easy and nice because there are some youths who feel shy to come to the clinic, so if the friendly corner was brought nearby, it can be better*”*.* However, young people still lacked sufficient agency to walk to the health centres to access contraception despite the use of peer educators and visiting the communities by peers was a welcome solution.

Respondent: the challenges are not there but some people are embarrassed that they will see me that I also come to get condoms. Others do not feel embarrassed, and it is very easy for them to get condoms.

Respondent: I would say that we used to face challenges but now it is different because the peer educators go into communities and people are educated from there. In the past people used to be scared of family planning. You know people are difficult some would say it is good and some would say it is bad. I would say information has spread in the community because of doing door-to-door visitation through role-plays.

[Adolescent males, low-performing rural district]

**Routine stock outs** prevented access to commodities for young people. In some rural health facilities in the low-performing context, there were stock-outs of both male and female condoms and contraceptive pills. The female adolescents reported that, apart from stock outs, there were no educational materials for adolescent peer educators to use when teaching their friends: Occasionally, the demand for condoms increased during special events such as weddings and other ceremonies, and the supply was insufficient. An adolescent male from a low-performing rural district said:

“No, the population here is big so we need more male condoms so that they are enough for everyone. Like here in the village in case there is a wedding in the neighbourhood village in the night we peer educators do carry condoms to distribute at the wedding to whoever wants them. The number of condoms should be increased”.

[Adolescent male from a low-performing rural district].

**The lack of dedicated time and spaces** for adolescent service provision was a common theme across all contexts. The lack of privacy left some of the adolescents feeling exposed to the rest of the community. This situation was worse for adolescents living with HIV as they sought refills for ARVs, which were done openly. One male adolescent from a low-performing rural district said: “*There isn't any privacy and when it's time to get the medicine you enter in a room for those getting and everyone else sees you when you enter that room. So some people feel shy*”*.* The COVID-19 pandemic compounded these challenges, as adolescents reported experiencing challenges with access to condoms because some of the interventions undertaken to improve access were abandoned. A female adolescent from a well-performing rural district said:

“Over the distance issue, they should be sending some workers in the field to make it easy for those far away to access the services. Like in February [2020], many school pupils wanted to have access to condoms so they formed a group and they used to come and educate us. But after the breakout of Covid-19, the groups were abandoned”.

[Female adolescent from a well-performing rural district].

#### Acceptability

##### Barriers to acceptability- contraception seen as linked to promiscuity

Although services were available in one way or another, dynamics were at play that could impede acceptability- the use of alternative medicine, the link between contraception and promiscuity, fear of infertility, religious views and being aware of reproductive health and rights. Adolescents narrated that there were herbal medicines that could be used to prevent pregnancy. A male adolescent from a rural low-performing district said: “(…) *the herbal medicines they use work in a way that a woman who gives birth is only given that medication after 6 months to prevent pregnancy in a short period before the other baby has grown.*” *A*dolescents from this context also noted the ease of interaction with fellow young people when accessing the services. A female adolescent from a rural low-performing district said:

“(…) when you come to the clinic you pass through first the peer educators, they counsel you then they take you to the doctor where there is privacy who tells us that the family planning, I have given you does not prevent diseases but pregnancy”.

[Female adolescent from a rural low-performing district].

##### Engaging community leaders shaped acceptability

However, even in the well-performing district, community leaders aimed to uphold their “good” reputation in their villages by regulating access to family planning services for adolescents and avoiding being labelled as supporters of prostitution. This was noted to restrict access to services for adolescents. In the urban low-performing district, the district health director reported the need to increase the acceptability of services in his region. When asked to provide an overview of the services in his district, he said:

“…I'll start with the adolescents, I think, we have about 6 health facilities which are championing the youth-friendly corner (…). To me, if we were to do well, I think if we were to cover everyone that would be much better because these youths are coming from all over [the district]. That is one of the issues to address concerning adolescents (…)

[District Health Director, urban low-performing district].

Community members from low-performing urban districts noted the link between family planning usage among women and promiscuity. The boys recalled being more accepting of their condom use, compared to the girls, -who were labelled as “prostitutes” when they tried to access the services or youth-friendly meetings. Young people said the community members felt that they were too young for family planning and sexual and reproductive health teachings. A young female from a low-performing rural said: “*Us* [the youth] *we are not supposed to get family planning because we will have that thought that* ‘*I am on family planning*’ *and start living careless and end up getting infected*”. A man from a rural well-performing district also said adolescents who accessed condoms and contraceptives were labelled as “loose” and indulged in careless sexual activities.

##### Fear of infertility as a barrier

It was common for adolescent girls from a low-performing facility in an urban district to avoid contraception until they had a child out of fear of contraceptive use-related complications and infertility when they finally decided to have babies in future. It was therefore warranted for healthcare providers to seek their parental consent before the provision of services, to protect their future fertility. Although this was the main justification, consent was also sought from partners of married women- creating a barrier to use. One male respondent advocated for partner consent by saying:

“(…) everything has precautions which we should follow and just as they said that if someone comes without having to consult their husband to say am going to do this, there might be a conflict in the house, so everything has an advantage and a disadvantage”.

[Adolescent male low-performing facility in an urban district].

In addition, future infertility was tightly woven with labels of promiscuity, as though it was the fate of all users of contraception. A female adolescent from a well-performing rural district said*:* “*(…) they usually discourage us by saying we will never conceive, saying if we use it, we will start having sex with different men as a result in the future will never bear children that is what they are saying in the community*”. Parents did not support the use of family planning.

##### Restriction of acceptability due to marital status

Family planning was only permissible for married community members, and adolescents and unmarried individuals were shunned for using this service. Women from low-performing urban districts debated the provision of contraception to young women and girls. One woman was not happy about sharing scarce commodities with young people, who were not even supposed to be using them. She said:

“Life is difficult, the life we are leading these days is hard, you can't give birth to a lot of children that my sister or my brother will keep for me, it finished that is long gone, you have to know the number of children you will give birth to (…). Why allow children to get family planning, they are allowing them to go and get HIV, because nowadays when we look, at long ago there were no children at family planning, they wanted those who are married They would even ask you, but nowadays why are you allowing school going children to be given family planning?”

[Adolescent female, low-performing urban districts].

##### Restrictions related to religious and traditional beliefs

While there was more access to contraception for young people, the community was not very happy about this. While very few religious sects or groups opposed the use of family planning, Christians were against the promotion of contraception use among the youth. A young man from the low-performing urban district in Muchinga narrated how the use of contraception was an act of going against his teachings as a person of Christian (catholic) faith because he knew he was not supposed to use contraception at all…

“(…) as Catholics we have discussions over such issues of family planning, and they teach us that Catholics are not allowed unless if they have seen that in having a lot of children they might risk their life… that is when they are allowed (…)”

[Young man from the low-performing urban district].

Another male adolescent from a low performing rural district said: “(…) traditional beliefs of the elders are that they should be an increase in population and hence they don’t encourage family planning”. Despite all these restrictions to acceptability and access barriers, young people mentioned that they were aware of their right to access services and the benefits of these services.

## Discussion

The findings of this study were described in the context of availability, accessibility, acceptability and quality of care of adolescents SRH services in selected provinces of Zambia. The AAAQ context helped to understand the value of a holistic approach to providing SRH services for adolescents in Zambia and similar settings (22) and taking into consideration the challenges and opportunities to provide interventions that translate into behaviour change (23). Generally, for the interventions explored in this study, efforts were made to increase the availability of services across the continuum of care and contexts. Peer providers were engaged to increase access to information and services, particularly the distribution of condoms and dedicated ART spaces (24). In the well-performing districts, community leaders were engaged to increase demand for these services and to encourage young people to go back to school. Such efforts were reported as effective, although the actual availability of trained sensitized and “youth friendly” healthcare providers and necessary commodities were not always available on demand. In addition, engaging community leaders to increase demand sometimes restricted access instead, due to the leaders’ other obligations in the community such as upholding the good behaviour of young people.

Therefore, it has been reported that what must be considered are young people's pathways to seeking services; and the specific barriers they face before getting to the services while receiving services, and after leaving the service delivery sites (25–27), which was observed and reported to be a challenge in the selected sites in this study. It has been recommended in similar settings to pay attention to the perceptions and needs of young people along with the development of policies, services, and programs that address these gaps in service delivery (25). Studies have indicated that attitudes can be changed through the implementation of diverse strategies, in this case, to effectively train healthcare providers in delivering adolescent-friendly health services that prioritize the individual. These strategies include values clarification training, relationship-building practice sessions, and the appointment of champions to drive transformative change (28).

Access to services was reported, as young people cited receiving information from schools, at the health facilities and within their communities- through a variety of actors such as teachers, healthcare providers, community members, and peer educators who were affiliated with health facilities. It was noted that well-performing contexts reported more activities while low-performance regions reported erratic provision of services, frequent stockouts, lack of trained healthcare providers and challenges with funding (29, 27). Thus, barriers to access were noted to lead to alternative sources such as herbal remedies and unauthorised contraception options which can be very unsafe (11).

Acceptability was generally low as most community members felt young people were not supposed to access these services, perpetually widening the “taboo gap” as suggested by Nesamoney et al. (30). In many African contexts, particularly in the sub-region, there is a prevalent unease when it comes to discussing sex-related topics with young people. This discomfort is often rooted in the fear of being misinterpreted as condoning or promoting sexual activity (31, 32). The absence of dedicated spaces and time for young people posed challenges to the delivery of services in certain contexts. Even when young people managed to access available spaces, they often faced the fear of being stigmatized as promiscuous, concerns about infertility, and apprehension about potential side effects. The fear of infertility poses a significant and longstanding barrier to contraceptive use, requiring specific attention to promote the uptake of contraceptive methods among young people (33, 34).

Consequently, many young people refrained from utilizing services, particularly contraception. Addressing this significant gap can be achieved by incorporating sexual and reproductive health (SRH) services within Safe Spaces, considering that young people are naturally drawn to these environments. By integrating SRH provision within Safe Spaces, the accessibility and attractiveness of these spaces can be enhanced for young individuals (35). Misconceptions about the efficacy of contraceptives and condoms have been linked to reduced acceptability and usage of contraception in other contexts too, without many interventions that target offsetting them (36).

Low acceptability also contributed to the rise in more private and acceptable alternatives such as herbal medicine. However, young people were aware of their right to use these services, but cultural norms and religious beliefs (37) contradicted these rights and reduced utilization. Other studies have suggested social accountability, as well as life skills-based education to respond to cultural barriers are suggested as potential solutions to increase the acceptability of highly stigmatized services, such as ASRH in most contexts, as suggested by this study (4, 38).

On the one hand, while the engagement of peer educators and community leaders were noted to increase the right of young people to access and utilize services, community values and beliefs continued to reduce this right, alongside other service-related challenges. On the other hand, it was also important to highlight the extent to which components of the interventions to increase SRH service utilization, according to Atun's framework, were shaped by the influence of: (i) the problem being addressed, (i) the intervention(s), (iii) the adoption system, (iv) the health system characteristics, and (v) the broad context—or integrated into routine adolescent health service provision and utilization.

It was a common perception that adolescents lacked access to services and faced challenges with availability and the low acceptability of adolescent health services, particularly, SRH services. A key indicator of these gaps is the high teenage pregnancy rates and negative health outcomes among young people. The interventions under discussion, AYFHS, were established in the early 90s, the system-wide intervention that has been rolled out and scaled up to all health facilities in the country (Adolescent Health Strategy, 2017–2021) (39).

According to the literature, the current funding intervention was rolled out at the district level in 22 districts of the southern and eastern provinces of Zambia, and all the 22 districts funded were non-randomly selected (13). However, the districts received the first funding disbursement meant for the initial implementation of the work plans in the fourth quarter of the first year due to some programme delays (13). While acknowledging that the Zambian health sector is hugely dependent on foreign assistance and external sources of funding, the delay in the disbursement of funds was significant (13). In addition, challenges with space accessibility, qualified human resources who may have positive or negative attitudes towards adolescents, and occasional stock-outs of commodities affected the implementation of these interventions. Feelings of lack of privacy, waiting times, and the lack of urgency when an individual is seeking information were cited as significant barriers to use, alongside fears of infertility, judgement, embarrassment and the lack of confidentiality and privacy, as corroborated by other studies (40–42). This points to some incompatibility of the interventions to the context.

Additionally, where young people failed to access or utilize services, alternative medicine was sought. In other places, these spaces have been modified, meeting under a tree, the use of peer educators under the supervision of trained HCPs. Also, the need to respond to increased demand during special occasions such as weddings and traditional ceremonies was mentioned by young people, like research findings on the need to adapt the provision of services during disruptions such as the COVID-19 pandemic (43). Some modifications to the interventions have proven effective in some contexts, while those that failed to innovate recorded poor health outcomes for young people. Social media interventions using the internet, newspapers or magazines, radio and television have proven effective in increasing demand for services (44, 45).

In consideration of the well-known service gaps in the health system, areas that had overcome shortages of trained, or even untrained healthcare providers still had challenges providing services and the quality of services was compromised. In addition, vast geographical areas are occasionally impassable. All these system-level barriers were also compounded by the COVID-19 pandemic, which restricted access to services and even healthcare providers as they were called to the frontline of the fight against the pandemic. Challenges of resilience during epidemics also question the extent of integration of AYFHS into the health system, suggesting more proactive consideration of these during system shocks (46).

### Strengths and limitations

It was noted that firstly, there were inadequate representations of all stakeholders, although provider and users' perspectives were captured, and triangulation of data sources and methods (using both interviews and group discussions) increased the credibility and dependability of findings. The study included three broad contexts-high performing, low-performing and one that had a mixed picture. In addition, two subregions for each of the three contexts were included, as well as variation in gender and age of respondents, and this increased transferability. While more detailed findings can be presented on the topic in future research, the description of the contexts is limited, but the description of the methods and procedures is much more elaborate, ensuring dependability and overall validity (47, 48). In addition, as this paper is based on a qualitative study, it adhered to the Standards for Reporting Qualitative Research (SRQR) to improve transparency of all aspects of the research, thereby increasing validity (49). Finally, this work was conducted by a specialist in maternal and child health, a specialist in women's health, a specialist in epidemiology and biostatistics and a specialist in adolescent sexual and reproductive health. All researchers understood the subject well and were all experienced in collecting, analysing and managing qualitative research data. Future research should more actively include more adolescents and other stakeholders in the sample size and should investigate the barriers to adolescent service provision using adolescent-friendly service guidance from the WHO. Additionally, more should be included on clinic funding disbursements related to SRH service provision, engagement of peers in increasing access to services, and engagement of community leaders.

## Conclusion

In addressing sexual and reproductive health from an SRH rights perspective, it is evident that progress has been made, although there is still much ground to cover. To bridge this gap and strengthen health systems, it is essential to focus on actionable recommendations that increase the acceptability of services across multiple actors. This comprehensive approach will significantly enhance service provision and utilization. One crucial recommendation is to foster multi-stakeholder collaboration, bringing together government agencies, NGOs, community leaders, and youth organizations. Through joint efforts, these stakeholders can work towards improving service acceptability and accessibility. Additionally, efforts should be made to secure regular funding, enabling sustained provision of services and overcoming barriers faced by some contexts.

Another critical aspect is addressing restricted access due to geographical distances. Contextually driven interventions tailored to specific areas can effectively reduce access barriers and improve service delivery. By implementing strategies such as mobile clinics, outreach programs, and telehealth services, healthcare can be extended to underserved regions. To further promote the SRH rights, it is important to engage in community engagement and education. This involves conducting awareness campaigns, educational programs, and community dialogues to combat stigma, dispel misconceptions, and garner support for youth-friendly services. Empowering young people through their active participation in decision-making processes and service design is also vital. By focusing on these recommendations, progress can be made in reducing access barriers, strengthening health systems, and upholding the human rights of young people in relation to their sexual and reproductive health.

## Data Availability

The raw data supporting the conclusions of this article will be made available by the authors, without undue reservation.
