# Supplementary material for: Understanding inequalities in the coverage of adolescent sexual and reproductive health services: a qualitative case study of the selected regions of Zambia
Source: Front Reprod Health. 2024 Aug 6;6:1399289. doi: 10.3389/frph.2024.1399289 (PMC11333446; doi:10.3389/frph.2024.1399289)
Supplement: Supplementary file 1 [file Supplementaryfile1.docx]

## Annexe 3.3: Discussion guide for Adolescents

| ***Selection Criteria:*** Must be male or female, age between 10 to 19 years, consent provided by a guardian for those below the age of 18 years, must be living within the communities at least for a year preceding the interviews. |
| --- |

Interviewer : ________________________________________

Date : ________________________________________

Location : ________________________________________

Safe motherhood No: ________________________________________

| **GUIDE TO MODERATOR**   - Greet the participant and thank them for accepting to be interviewed. - Copies of informed *consent* forms should be provided to the participants and read aloud for the benefit of those who cannot read. - Introduce yourself as an interviewer and ensure you familiarize with the participant. - Explain about use of the study outcomes. - Complete the demographics form with the participant. - Verbal agreement should be audio recorded. - Try to ask all the questions below in the order given, but it is more important to maintain the flow of discussion. - Suggested probes have been included. - The participants should be provided an opportunity to ask any questions.   *Before we start I would like to remind you that there is no right or wrong answers in this discussion. I am interested in knowing what you think, please feel free to share your views, regardless of whether you agree or disagree with what you know.*  ***Interviewer should introduce herself/himself and explain the purpose for the interview.*** |
| --- |

**Introduction**

Good morning/afternoon. My name is …………………………………… `Thank you for showing interest in this study. I hope you have understood the information provided before deciding whether or not to participate. If you decide to withdraw from the interview, that is fine. There will be no disadvantage to you and we thank you for considering our request. Before we start, I would like to remind you that there are no right or wrong answers. We are interested in knowing what you think, so please be free to share your views. It is important that we hear all your opinions.

| **Availability, accessibility and acceptability of family planning services among adolescents** | |
| --- | --- |
| Availability of family planning and contraception services | *Let’s start by talking about your views on the family planning services being offered at the health centre.*  Could you tell me what your understanding is about family planning services being provided at the health centre?  **Probe:** Are you are aware of the services being provided?  **IF YES,** tell me how you heard about these services  **Probe:** Has a healthcare worker or anyone else told you about family planning services? IF NO tell me more about where you heard about it  **Probe:** Tell me what you know about the different family planning services that you know and are being provided at the health facility  **Probe:** Tell me how you heard about the care, where did you hear about this care?  Which type of family planning services do you know that are being provided at the health facility  **Probe:** What do health care providers or other personnel in the facility do to provide this service?  **Probe:** Counselling  **Probe:** Other?  **Probe:** are there services that you want to receive but are not available?  Have you ever received family planning services from the health centre?  IF YES, could you tell me which family planning services you received at the health centre  **Probe:** Type of family planning  **Probe:** Who provided the service |
| Acceptability of family planning services | *Now tell me more about your views on the kind of family planning services being provided at the health facility*  What are your thoughts about the kind of family planning services being provided at the health centre  **Probe:** What do people in your community say about immunisations?  **Probe:** Religious beliefs related to immunisations  **Probe:** Cultural Beliefs related to immunisations  **Probe:** Environment  **Probe:** Attitude of health care providers  **Probe:** Gender preference of health care provider  Now tell me about the service itself, do you feel that you are getting the care that you expect? Tell me more about it  **Probe:** Available equipment for tests  **Probe:** Available treatment and supplies  **Probe:** Available health care providers  **Probe:** Trust in the skills and competence of the health care providers  Do you have any concerns about the family planning services? Tell me more about it  Would you recommend a colleague to seek family planning services at the health centre? Tell me more about your response  **Probe:** Why would you/why would you not?  What do you think could be done well about in the way family planning services are provided in at the health centre? |
| Accessibility of family planning services | *Now, tell me how it is like to get the family planning services from the health centre*  Does anything make it hard for you to come to this clinic for family planning services? **IF YES**  What made it hard for you to receive family planning services from the health centre?  **Probe:** Too far to travel to come to the clinic to get the care you needed?  **Probe:** Too expensive to pay for transport or such as bicycle taxi?  **Probe:** Cost of services too much? How much do you spend?  **Probe:** You feel too embarrassed to seek the services?  **Probe:** Not enough privacy?  **Probe:** Was it because you couldn’t take time away from work or taking care of children, or other household responsibilities?  **Probe:** Were you afraid that other people (friends, family) might find out  **Probe:** Because the health care workers are not supportive (do not explain things, did not help you in the way you wanted or expected?  **Probe:** How are you treated when you go to the health facility? Do you know anyone? What happened?  What made it or would make it easier for you to come to the clinic for family planning services  **Probe:** Would having it in another location closer to your home/village make it easier? |
| **Availability, accessibility and acceptability of STI and HIV/AIDs services among Adolescents** | |
| Availability of STI and HIV/AIDs services | *Now that we have discussed your views on the family planning services that are available and what it means to get the services, let us now focus on your own views on HIV and STI services.*  Have you ever heard about diseases known as STIs? If yes?  **Probe:** Tell me what you have heard about it?  **Probe:** Tell me how you came to know about this disease  Have you ever heard about a diseases known as HIV/AIDs If yes?  **Probe**: Tell me what you have heard about it?  **Probe:** Where have you heard about the disease  Now let’s talk about the care that is supposed to be provided at the health centre for STIs and HIV/AIDs  Do you know if this care is available at the health facility?  **IF YES,** how did you get to know about the care being provided?  **Probe:** Tell me what you know that is being done at the health facility for people that come to seek help for STIs  Have you ever sought care for STIs or HIV/AIDs from the health centre? IF YES, please tell me which specific disease  What did health care providers or other personnel in the facility do to provide this care  **Probe:** Counselling  **Probe:** Testing  **Probe:** Treatment  **Probe:** Return of results  **Probe:** Interpretation of results |
| Acceptability of STIs and HIV/AIDs services | Can you tell me more about the services provided at the health centre for the prevention of STIs/HIV/AIDs? What are your thoughts about the kind of care  **Probe:** Now tell me about the care itself, do you think that you are getting the care that you expect? Tell me more about it  **Probe:** Available equipment to test for STI/and HIV  **Probe:** Available treatment  **Probe:** Available health care providers  **Probe:** Tell me more about the environment the care is being provided.  **Probe:** Privacy  **Probe:** Confidentiality  **Probe:** Waiting time  Do you have any concerns about the services? Tell me more about it  What do you think could be done well with the services being provided in the health centres?  **Probe:** Now tell me about the care itself, do you feel that you are getting the care that is appropriate? Tell me more about it  **Probe:** About the health care providers |
| Accessibility of STI and HIV/AIDs services | *Now tell me about your view or other young people’s views about STIs/HIV/AIDs services*  What are some of the challenges that you have faced to access the services you have just indicated  Does anything make it hard for you to come back to this clinic? **IF YES**  What made it hard or might make it hard for you to come back to have your baby HIV tested?  **Probe:** Too far to travel to come to the clinic to get the care you needed?  **Probe:** Too expensive to pay for transport or such as bicycle taxi?  **Probe:** Cost of services too much? How much do you spend?  **Probe:** You feel too embarrassed to seek the services?  **Probe:** Not enough privacy?  **Probe:** Was it because you couldn’t take time away from work or taking care of children, or other household responsibilities?  **Probe:** Were you afraid that other people (friends, family) might find out  **Probe:** Because the health care workers are not supportive (do not explain things, did not help you in the way you wanted or expected?  What made it or would make it easier for you to come back for the services for prevention of STs/HIV/AIDs?  **Probe:** Would having the services in another location closer to your home/village?  **Probe:** Why  **Probe:** More possibilities |
| **Availability, accessibility and acceptability of youth friendly spaces among Adolescents** | |
| Availability of youth friendly corners | Now I would like us to look at the spaces that are available within the health facility that specifically attend to young people like you  Have you ever heard of any of such spaces within the health facility that focus on care for young people?  **Probe:** Where did you hear about such spaces  **Probe:** Have you ever heard of a youth friendly corner? If yes tell me more about it?  **Probe:** What services are provided in such spaces  **Probe:** Describe the most ideal youth-friendly space.  **Probe:** what services could make you want to go there?  Now tell me, have you or anyone you know ever sought care from the youth friendly corner?  **Probe:** Tell me about the providers of the services in such spaces  **Probe**: Is the environment conducive (privacy, confidentially, stigma)  **Probe**: Would having the services in another location closer to your home/village?  **Probe**: Why  **Probe:** What do you want them to do? |
| Acceptability of family planning services | Now tell me about your thoughts or other young people’s views of the of youth friendly facility that is there.  Is there anything you would like to share that that is of your concern regarding the youth friendly corner in the health facility  **Probe**: What do people in the community say about these youth friendly spaces?  **Probe**: What traditional beliefs do you know that affect your uptake of family planning and contraception?  **Probe**: What religious beliefs do you know that affect your uptake of family planning and contraception?  **Probe**: What about the providers? Do you trust them?  **Probe:** Attitude of health care providers  **Probe:** Gender preference of health care provider |
| Accessibility of family planning services | What are some of the challenges that you have faced to access the services from the youth friendly corners.  Does anything make it hard for you to come back to this clinic? **IF YES**  What made it hard or might make it hard for you to come back to have your baby HIV tested?  **Probe:** Too far to travel to come to the clinic to get the care you needed?  **Probe:** Too expensive to pay for transport or such as bicycle taxi?  **Probe:** Cost of services too much? How much do you spend?  **Probe:** You feel too embarrassed to seek the services?  **Probe:** Not enough privacy?  **Probe:** Was it because you couldn’t take time away from work or taking care of children, or other household responsibilities?  **Probe:** Were you afraid that other people (friends, family) might find out  **Probe:** Because the health care workers are not supportive (do not explain things, did not help you in the way you wanted or expected?  What made it or would make it easier for you to come back for the services at the youth friendly corner |
| **Closing Remarks** | 1. Is there anything else you would like to share that we did not already discuss today? 2. Is there any care that you think can be provided better among what we have discussed? 3. Tell do you think could be done better 4. Do you have any questions for me? 5. Thank the participant for time and input and assure the sharing of study findings with participants. |
| Demographic Data | 1. How old are you?   …………………..   1. What is your Sex?   Male ……….1  Female………2   1. Are you currently married   Married …………………………1  Not Married but have a partner….2  Not married and no partner ……..3  Separated/divorced ……………..4  Widowed…………………..…….5   1. What is your level of education   Never been to school …………1  Some Primary school …………2  Completed Primary School ……3  Some secondary school ……….4  Completed Secondary School….5  Tertiary level …………………6 |
